# Recombination rate plasticity: revealing mechanisms by design

**DOI:** 10.1098/rstb.2016.0459

**Published:** 2017-11-06

**Authors:** Laurie S. Stevison, Stephen Sefick, Chase Rushton, Rita M. Graze

**Affiliations:** Department of Biological Sciences, Auburn University, Auburn, AL 36849, USA

**Keywords:** recombination, *Drosophila*, crossing-over, meiosis, oogenesis, plasticity

## Abstract

For over a century, scientists have known that meiotic recombination rates can vary considerably among individuals, and that environmental conditions can modify recombination rates relative to the background. A variety of external and intrinsic factors such as temperature, age, sex and starvation can elicit ‘plastic’ responses in recombination rate. The influence of recombination rate plasticity on genetic diversity of the next generation has interesting and important implications for how populations evolve. Further, many questions remain regarding the mechanisms and molecular processes that contribute to recombination rate plasticity. Here, we review 100 years of experimental work on recombination rate plasticity conducted in *Drosophila melanogaster*. We categorize this work into four major classes of experimental designs, which we describe via classic studies in *D. melanogaster*. Based on these studies, we highlight molecular mechanisms that are supported by experimental results and relate these findings to studies in other systems. We synthesize lessons learned from this model system into experimental guidelines for using recent advances in genotyping technologies, to study recombination rate plasticity in non-model organisms. Specifically, we recommend (1) using fine-scale genome-wide markers, (2) collecting time-course data, (3) including crossover distribution measurements, and (4) using mixed effects models to analyse results. To illustrate this approach, we present an application adhering to these guidelines from empirical work we conducted in *Drosophila pseudoobscura*.

This article is part of the themed issue ‘Evolutionary causes and consequences of recombination rate variation in sexual organisms’.

## 1. Introduction

Understanding biotic and abiotic influences on genetic variation in natural populations is a central goal of evolutionary genetics. The two main processes that contribute novel genetic variation to a population are mutation and recombination, both of which have been shown to be influenced by environmental processes [1]. Changes in recombination rate can pose both benefits and complications. For example, there is much theoretical work on the evolution of sex that discusses the benefits of meiotic recombination in facilitating selection to novel environments [2–5]. Indeed, facultative asexual organisms switch to a sexual life cycle in stressful conditions, presumably for the benefits of meiotic crossing-over [6]. However, meiosis is tightly regulated and alterations in crossing-over have the cost of increased rates of nondisjunction, which can lead to aneuploidy [7–9]. For example, trisomy 21 is suggested to be largely due to recombination abnormalities specifically resulting from maternal problems in the first stage of meiosis (see more below) [10]. Similarly, several human cancers are associated with abnormal mitotic recombination, including breast and ovarian cancers [11]. The well-known BRCA1/2 mutations are sensitive to environmental perturbations, such as ionizing radiation [12].

It is important to understand the specific mechanisms contributing to sensitivity of recombination rates to the environment and how it may impact both the health of individuals and the overall evolution of species. Recombination rates are conserved between closely related species when measured in intervals greater than 500 kb [13,14]. By contrast, studies in great apes have shown that recombination rates evolve more rapidly than corresponding nucleotide changes [15]. Thus, environmental sensitivity allows single generation changes in recombination rate without corresponding changes in nucleotide substitution. Further, natural genetic variation in recombination rate has been shown to vary up to twofold [16], whereas environmental heterogeneity leads to three- to four-fold changes in recombination rates in *Drosophila* [17,18]. Much speciation research has shown that changes in recombination between species can drive divergence and facilitate speciation [19]. In a study in *Sordaria*, higher recombination rates were found in a population from a more extreme environment when compared with a nearby population in a milder environment [20].

Recombination rate differences due to environmental or physiological differences are often referred to as recombination rate ‘plasticity’ in the literature. The term *plasticity* has different meanings in other fields (for discussion see [21]). For clarity, we use it here to match literature referring to differences in observed recombination rates associated with various environmental, physiological or stressful conditions. Classic research in this area grew from linkage studies in the model organism *Drosophila*. The earliest studies explored *Drosophila* recombination rates in a wide variety of experimental treatments, with varying degrees of exposure, including age of the mother, starvation, extreme temperatures and humidity levels [22–26]. For example, the effects of temperature stress (e.g. rearing flies outside of their normal thermal range) and their impact on broad-scale recombination rate variation were examined using different exposure times and marker pairs in multiple regions of the genome [24]. In general, recombination rates increase owing to these selected treatments [1,18]. Other model taxa, such as yeast, *Caenorhabditis elegans* and *Arabidopsis*, have a long history of studies on recombination rate plasticity [27–30]. Studies outside of model taxa have been mostly restricted to plants and fungi, likely owing to the ease of genetic manipulation. Additional studies in other taxa include mouse, human, tomato, grasshopper, tobacco, maize, *Coprinus* and *Sordaria* [20,31–39]. Many of these studies have used stressful conditions similar to *Drosophila* experiments (e.g. temperature, age, starvation, irradiation and pathogen stress).

Here we summarize representative studies in *Drosophila*, illustrating how these studies support several possible mechanisms, and connect the body of work in this key model to work from other taxa. We also explore how broadening the number of species in which recombination plasticity has been studied will potentially resolve outstanding questions and provide new insights. The knowledge gained from approaches developed in *Drosophila* is widely applicable, with modification, based on specific knowledge of meiosis and gametogenesis in the organism under study.

In this review, we consider those studies that test mechanistic hypotheses explaining recombination rate plasticity. Recombination and mutation both occur at very low per site frequencies (approx. 10^−5^ and approx. 10^−9^, respectively [40,41]). This makes detection of experimental differences challenging, and requires large experiments with thousands of individuals. Plentiful phenotypic mutant markers in *Drosophila* have made it possible to measure recombination rate differences *en masse* without molecular resources [18]. For other model systems, such as yeast, metabolic screening has been used to facilitate rapid phenotypic screens for recombinants [29]. Additionally, *Arabidopsis* studies have used reporter transgenes to assay recombination rapidly [27]. Now, with the development of widely applicable sequencing and genotyping techniques [42–44], and genome editing tools [45,46], the approaches developed in model organisms can be easily adapted for use in non-model organisms. Combining classic methodology with modern technological advances may also allow breakthroughs in understanding the mechanistic basis of recombination rate plasticity, an area that is relatively understudied. However, without experimental designs that pinpoint the broad underlying mechanisms (e.g. crossover control), uncovering the genes and networks that contribute to recombination rate differences is challenging. Synthesizing early work in the field, and incorporating new developments, we present guidance on how to meet this challenge and illustrate our own application of these approaches with data using molecular markers we designed in *Drosophila pseudoobscura*. While this is a classic system used for population genetics [47], it lacks the suite of mutant markers and other genetics resources available in *Drosophila melanogaster*, and, therefore, the approaches we use here will apply to other non-model systems. We hope that highlighting the lessons learned from 100 years of studies in *Drosophila* (including the preliminary findings we describe within) will encourage scientists to apply these simple, yet powerful, methods to the study of recombination rate plasticity in other systems.

## 2. Gametogenesis, meiosis and recombination in *Drosophila*

In 1915, at the dawn of genetics, Calvin Bridges found that the linkage maps constructed from observing many hundreds of crossover events in individual fruit flies could change over time [25]. The measurements of the amount of crossing-over, which formed the foundation of the earliest understanding of the physicality of genes and their locations, were a moving target. Measurements made from progeny of younger females showed a higher percentage of crossing-over (i.e. longer recombination distances between genes) than did those calculated from the same females as they aged [26]. A literal interpretation would have been that, as animals aged, genes changed in position. Bridges, considering what was known at the time about the mechanisms of recombination, concluded that there were two possible explanations. The number of recombination events could be changing owing to direct changes in DNA breakage and repair, or the ‘tightness of the twists’ of a chromosome could be changing with age and affecting interference. This simple mechanistic question of what causes recombination rates to be variable for the same individual or genotype (i.e. recombination plasticity) is still unresolved.

Since the foundational studies of Bridges, there has been extensive study of recombination in *Drosophila* owing to the ease of rearing animals in the thousands, making it the core model for both evolutionary [16,18,48–51] and molecular genetics of recombination [10,52–56]. These studies include both classic studies using visible morphological markers or features of chromosome structure [16,22–26] and recent studies based on molecular markers [50,57,58]. At the same time, and for similar reasons, *Drosophila* became the core model for the genetics of gametogenesis and meiosis. Studies of recombination and meiosis in this system are facilitated by visualization of chromosomes using straightforward cytogenetic techniques. Progress in the genetics of recombination in *Drosophila* has also been greatly aided by extensive genome annotation, sophisticated transgenic tools and a scientific culture of resource sharing [59–62].

**Figure 1.**
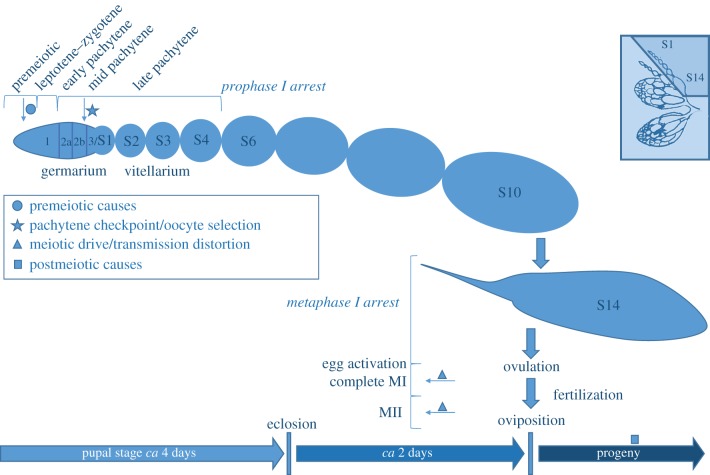
Oogenesis, meiosis and mechanisms of recombination rate plasticity. The mature ovary (top right; adapted from Miller (1950) [63]) consists of multiple ovarioles. Multiple stages of oocyte differentiation are present in each ovariole present in the adult female. Details are shown for a single ovariole (shaded region of ovary) during the first round of oogenesis within the mother, to illustrate how these initial stages may be perturbed during development. Selected events during meiosis are shown to provide context. Time points of possible mechanisms of recombination rate plasticity are highlighted (filled symbols).

Studies in *Drosophila* have revealed both the phenomenon of recombination rate plasticity and possible mechanisms that explain sensitivity of recombination rates to physiological and environmental perturbation. An understanding of the timing of events in meiosis and gametogenesis is critical for designing and interpreting these types of experiments. To provide a framework for how mechanisms of recombination rate plasticity are elucidated using perturbation or time-course-based experimental designs, we provide an overview of meiosis and gametogenesis, focusing on the *Drosophila* oogenesis model. In *Drosophila*, oogenesis takes approximately 6 to 7 days to complete from the first mitotic divisions to egg activation, fertilization and deposition (figure 1). Gamete production begins during development before eclosion of the adult animal; eggs and sperm are continuously produced throughout adulthood. Meiotic recombination occurs only in female *Drosophila*. In males, meiosis occurs via an alternative achiasmate pathway [64,65].

Meiotic recombination plays a critical role in sexual reproduction, contributing to correct segregation of homologous chromosomes and variability among offspring (see [56] for review). The two divisions of meiosis, a reduction division (MI) going from diploid to haploid, and an equational division (MII) separating sister chromatids, are organized into defined stages (prophase, metaphase, anaphase, telophase and cytokinesis). Direct environmental effects on recombination are expected to occur either pre-meiotically or during the extended prophase of MI. As shown in figure 1, the critical stages with respect to recombination within prophase I are leptotene (condensation, organization and double-strand break formation), zygotene (pairing and synaptonemal complex formation) and pachytene (crossing-over).

Differences in gametogenesis and timing of meiotic events across taxa can be extensive, and this timing will alter when direct or indirect effects on recombination rates may occur during the life cycle. Meiotic arrest is one mechanism that allows coordination of meiosis with other aspects of gametogenesis, gamete release and fertilization. The precise timing of meiotic events and the number and duration of meiotic arrests are often sex and species specific. For example, progression of meiosis during oogenesis in animals is coordinated with ovulation and/or fertilization. In *Drosophila*, prophase I continues over multiple days in developing egg chambers within the vitellarium (figure 1). Meiosis then arrests at diplotene, during stages 5–8, [66]. In contrast, the prophase I arrest in mammals occurs during early development and is maintained for many years prior to ovulation. Despite these differences, studies in both model species and non-model species inform one another; the temporal regulation of meiosis has similar properties in all animals and the stages and arrests are directly comparable [67].

In *Drosophila*, oocytes are visible at progressive stages of development in adult animals, from the earliest stages of meiosis to the formation of mature eggs (figure 1). *Drosophila* ovaries are made up of many individual ovarioles (figure 1). Each ovariole contains a series of egg chambers at progressively more mature stages [68,69]. The first ovarioles form during the transition from the larval to pupal stage. Located at the anterior end, each stem cell of the germarium will divide asymmetrically; one daughter cell maintains stem cell identity and the other differentiates into a cystoblast. The cystoblast then divides mitotically to produce a single 16-cell germline cyst, visible in region 2a of the germarium. Pre-meiotic S-phase (DNA replication) occurs at this early time point in all 16 cells [70]. Subsequently, several cells within a cyst may initiate prophase I of meiosis, although only two become pro-oocytes [71]. At the transition between regions 2a and 2b, and concurrent with the pachytene checkpoint, a tightly regulated oocyte selection process occurs and only a single oocyte will progress through subsequent stages of meiosis [72–74]. In other higher eukaryotes, such as in mammals, multiple cells that make up a germline cyst can develop into oocytes. Interestingly, while no formal process of oocyte selection is recognized in mammals, only about a third of cells in the cyst will go on to become primary oocytes [75]. This means that the process by which either single or multiple oocytes are selected could affect realized recombination rates both in *Drosophila* and in mammals. In general, direct effects are expected to occur during these early meiotic stages. Specifically, recombination rates are sensitive to perturbations just before or during pachytene when both crossing-over and oocyte selection occur.

Indirect effects on the realized recombination rate could also happen during later stages of meiosis, specifically during the MI and MII divisions. Egg chambers progress through many stages of development between early meiosis and these later divisions. In fact, progression from prophase to metaphase I can take approximately 5 to 6 days [76,77]. Metaphase I does not occur until stage 14 and is closely followed by a second arrest. Metaphase I arrest is released upon egg activation during ovulation [67]. Meiosis then completes over the next 48 h with the MI and MII divisions occurring concurrently with fertilization and egg deposition. Transmission distortion at these stages could alter the realized recombination rate [78].

**Figure 2.**
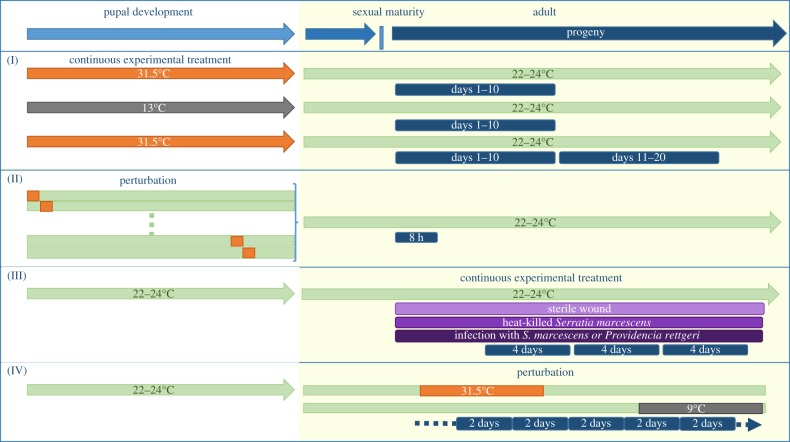
Temporal and developmental elements of experimental design. The timing of the experimental treatment and brood collections is diagrammed for each type of approach, highlighting the example discussed. (I) Continuous exposure during development. Example: Plough 1917 [22]. F_1_ females are reared under experimental conditions throughout development (including the pupal stage, white background) and returned to control conditions after eclosion (during sexual maturation and adulthood, yellow background). One or more broods are collected (navy blue). (II) Perturbation during development. Example: Grell 1971 [23,80–83]. A series of perturbations in experimental conditions are conducted. Each perturbation (orange) covers a defined window of time during pupal development. Only the first 10–15 eggs, the first egg maturing from each ovariole, are assessed for recombination rate. (III) Continuous exposure in adult stages. Example: Singh *et al.* 2015 [84]. Experimental treatments affect physiological conditions of the adult female at the same time as progeny are collected. Mature females were infected with pathogenic bacteria (dark purple), and compared with control treatments (light purple). Multiple broods were collected concurrently with the treatment effects. (IV) Perturbation during adult stages. Example: Plough 1917 [22]. Controlled perturbations are applied during adulthood, with progeny collected before, during and after the change in experimental conditions. In all cases, broods from genetically matched control F_1_ females reared in standard room temperature conditions were also collected (not shown).

The processes that can affect recombination rates are distinctly separated in time as oogenesis proceeds through subsequent stages. This timing allows simple experimental designs in *Drosophila*, and other systems, which distinguish between two broad mechanistic hypotheses. Specifically, the timing of changes in recombination rates can distinguish between early meiosis or pre-meiotic stages, and late or post-meiotic stages. For example, some organisms undergo an inverted form of meiosis [79], with reversed timing of MI and MII, which would be uniquely useful for distinguishing between indirect effects occurring during these divisions. In general, the logic of how mechanistic inferences are made depends on the experimental approach and the system under study. Recombination rate plasticity is expected only if a developing gamete is subject to stressful conditions at the time a causal mechanism is occurring. For example, in *Drosophila* only oocytes at early stages of development are present prior to eclosion. If an experimental treatment is applied during development and removed post-eclosion, the hypothesis that causal mechanisms occur in these early stages of meiosis can be tested. To clearly explain how the interpretation of the data differs between different approaches we highlight a specific example for each basic type of experimental approach (figure 2), and relate these experiments to oogenesis and meiotic mechanisms suggested by the results (figure 1).

## 3. *Drosophila* as a model for studying recombination rate plasticity

Common elements of experimental design are shared across most existing studies of recombination rate plasticity. To test for effects of an environmental condition, heterozygotes are exposed to an experimental treatment and compared with genetically matched controls reared in standard conditions. In *Drosophila*, recombination studies are always done with females because there is no meiotic recombination in males. Treatment and control females are backcrossed to one of the two homozygous parental strains and allowed to lay eggs for a number of days. Progeny of each female are scored using visible or genetic markers and recombination rates are calculated. Data may be analysed for either a single laying period all together, separately for progeny from each day, or in several sets, each grouped across multiple days. Although *D. melanogaster* females lay continuously, each group of progeny is considered separately and referred to as a brood. The temporal relationship between application of the treatment and the observation of recombination rate plasticity in the progeny is a critical indicator of the timing of causal events. Pre-meiotic stages of oogenesis, meiotic stages and post-meiotic stages occur in succession over many days (figure 1). The distance in time between a causal event and observation of recombination rate plasticity in the progeny is indicative of when the event occurred during oogenesis. Different mechanistic hypotheses result in testable predictions of how recombination rate plasticity changes over consecutive broods of progeny. Within the framework of this basic design, there are different ways that experimental treatments have been applied. For example, in many studies in *Drosophila*, and in other taxa, heterozygotes are exposed to the experimental treatment continuously during development, while other experiments apply treatments in a series of developmental perturbations [23,80–82]. Additionally, plasticity has been examined using continuous exposure in adult animals and perturbation in adult animals (figure 2).

### (a) Possible mechanisms occurring at pre-meiotic stages or during early meiosis

Among the earliest studies of recombination plasticity are those conducted in 1917 by Plough [22], which measured the percentage of recombinant progeny after exposure to a variety of experimental treatments (figure 2(I)). Plough's experiments used sets of visible markers and a Mendelian cross design. Starting from the cross between parental strains homozygous for wild-type or recessive visible mutations (*black*, *purple*, *curved*; *b-pr-c*), crosses were reared during development with F_1_ females collected either after exposure to treatment or from control conditions. These F_1_ females were then backcrossed, in control conditions, to the *b-pr-c* parental strain males upon reaching sexual maturity and allowed to lay eggs for 10 days. The percentage of recombinant progeny was calculated over all 10 days (a single 10-day brood). Many of the experimental treatments investigated had no significant effect, but striking increases in recombination rates were observed when exposing females to extreme cold or extreme heat, when compared with room temperature control conditions, indicating substantial plasticity (figure 2).

To test for temporal effects that might indicate the underlying mechanism, consecutive broods of progeny were collected in the next set of experiments. The difference in recombination rate between treatment and control was compared over time. By examining the length of time that the effect persisted after the females had been returned to control conditions, it was found that differences were limited to approximately the first 7 days of laying. This coincides roughly with the time it takes for a *D. melanogaster* egg to complete oogenesis (figure 1). Plough [22] concluded that the effect of temperature was limited to a single early point during oogenesis. All developing oocytes that exist in the mother during the pupal stage pass through this point at high temperature and, therefore, the eggs deposited by the female show plasticity until all of these oocytes have reached maturity. This approach is useful to test for early mechanisms, but cannot clearly distinguish between different events during early meiosis.

A later set of studies conducted in 1965–1978 by Grell [23,80–83] addressed this issue. High-temperature perturbation was used as a tool to determine the timing of finer-scale meiotic events in early oogenesis. The goal of these studies was to determine when pre-meiotic S-phase and recombination occur during oogenesis and to precisely define the developmental time points at which temperature shifts recombination rates. The key innovation was an experimental design in which synchronized populations of oocytes could be exposed to a heat treatment (35°C), and the effect assayed accurately in the first 10–15 eggs deposited by each female. By focusing on the initial round of oogenesis occurring during development of the ovary, temperature perturbations of the earliest stages of oogenesis could be tested separately from perturbations during later stages. Experiments with increasingly narrow time windows of temperature perturbation, from 24 to 6 h, were used to define a ‘critical period’ during which oogenesis was most sensitive to temperature changes.

Considering the timing of events in the initial stages of meiosis present in the developing female, the results from the work of Plough and Grell are consistent with temperature-induced changes in recombination occurring early in meiosis. Under constant experimental treatment during development, all the eggs in the developing females are exposed to the experimental treatment at this sensitive time point and, as expected, only oocytes collected in the first 7–10 days show changes in recombination rates. If temperature is perturbed in the adult for approximately a week, recombination rates rise in the progeny of treated females just after the treatment is ceased and this is maintained for an approximately equal period of time, before dropping to control levels. The detailed studies of heat perturbation during development conducted by Grell are also consistent with an early meiotic effect. This work identified a period of approximately 36 h—starting at a time at which only very early pre-meiotic stages would be present in most females and extending into the time period of pro-oocyte differentiation and oocyte selection. Temperature perturbations before this time period and those after it, but pre-eclosion, had little to no effect.

Early studies in *Drosophila* [81], and in other species (discussed in [85,86]), suggest early meiotic, and possible pre-meiotic mechanisms. While these early studies laid the foundation for understanding recombination plasticity, they pre-date molecular genetic knowledge of meiotic recombination. For example, oocyte selection takes place concurrently with the pachytene checkpoint, with errors causing a delay in oocyte selection [72,74,87]. Thus a competitive mechanism could result in higher apparent recombination rates [88], if pro-oocytes destined to have a higher rate of crossing-over preferentially differentiate into the oocyte. Molecular mechanisms of recombination rate plasticity are not well understood and have not been extensively studied. It is, therefore, unclear if effects of environmental perturbation at early meiotic stages affect the regulation of crossover events (hereafter CO) or affect CO formation directly. Studies in plants and yeast have shown that there are potentially direct links between regulators of stress response and crossover formation (reviewed in [89]). For example, stress response genes in yeast directly regulate the amount of recombination at the location of recombination hotspots [90,91]. Other studies suggest the possibility that environmental perturbation modulates regulation of CO events. These events and subsequent stages of early meiosis are nearly simultaneous and may not show distinct temporal signatures for different possible early mechanisms. In *Drosophila* the cells in a cyst show pre-meiotic chromosome organization and pairing, with synaptonemal complex present at centromeres, before mitosis is complete [92,93]. It is possible that environmental effects at these very early stages could impact recombination if these processes are necessary for later meiotic pairing and synaptonemal complex formation.

Experimental treatments applied in the adult stages can also be used to test if processes that occur later in oogenesis explain apparent plasticity in recombination rates. Plough [22] explored the effects of perturbations during adult stages (figure 2 (IV)). To do this, F_1_ females were reared to maturity and backcrossed under control conditions. Two-day broods were collected continuously for each female, until no more progeny were obtained. Control females were maintained at room temperature during adulthood, and experimental treatment females were subjected to high or low temperature. The results revealed a multi-day delay between application of the treatment and a plastic increase in recombination rates, consistent with early stage processes. Thus, this approach allows for a broader understanding of possible mechanisms based on both how long it takes for plasticity to manifest, and the duration over which plasticity is observed. These temporal effects must then be matched to events in oogenesis and meiosis.

### (b) Possible mechanisms occurring late in meiosis or after meiosis is complete

Other studies have supported mechanisms that affect the realized recombination rate at later stages of gametogenesis, after recombination has already occurred. The first set of experiments in recent work of Singh *et al.* [84], applied treatments in the adult stages, similar to the adult temperature perturbation studies of Plough. The common elements of experimental design were used as discussed previously. F_1_ females developed and eclosed under normal conditions, but mature females were infected with pathogenic bacteria. Recombination rates in progeny from infected females were compared with those from a series of controls (figure 2(III)). An initial experiment, examining a single 5-day brood, found recombination rate plasticity due to infection. The experiment was repeated with progeny collected from two 4-day broods. Increased recombination rates due to infection were observed in both the first and second broods.

With this type of approach, a delay in the observation of recombination rate plasticity is expected if the causal events occur during the earliest stages of oogenesis. This is because effects that occur during the earliest stages of oogenesis will not be observed until the entire process is complete and eggs are deposited. Thus, the ‘early meiosis’ hypothesis predicts a significant change in recombination rates only in the second brood or an increase when comparing the first and second brood. In contrast, if the mechanism occurs late in meiosis or post-meiotically, during egg maturation and deposition, an immediate effect resulting in plasticity in the first brood is expected. The results, therefore, indicate that processes occurring in later stage meiosis, post-recombination, have shifted the observed recombination rate [84].

During these later stages a second potentially non-random competitive event occurs [94–96]. This latter process has been theorized to explain most transmission distortion [84]. During this time, only one in four gametes survives MII, becoming the pronucleus, while the rest become polar bodies [96]. If selection of the pronucleus is not random with respect to recombination, apparent differences in recombination rate may be observed. The molecular mechanisms that might link recombination and designation of the pronucleous are entirely unknown. Further, post-meiotic effects on viability could also contribute to apparent transmission distortion and plasticity in recombination rates, which represents an interesting and active area of research [78,97]. While segregation distorters have received much attention [98–100], the genetic basis of recombination rate differences introduced by transmission distortion is likely to be complex and there is currently little insight into the genes and networks that may govern these processes.

### (c) Other possible mechanisms

In some cases, such as for severe starvation [101] and parasitic infection [84], recombination rate plasticity is observed, but the change is maintained over the lifetime of the female. This is consistent with permanent effects of stress on the stem cell niche or germline stem cells themselves. Stress can dramatically affect the process of oogenesis in females [102]; for example, lack of a rich protein source results in reduction of daily egg production from 90 to 1.5 eggs. Decreased egg production occurs due to slower germ cell development and apoptosis of stage 8 nurse cells, resulting in degenerate ovaries. The effects on oogenesis are dramatic and may indirectly affect recombination rates, given the occurrence of slower progression and apoptosis in the 2a–2b region of the germarium in which oocyte selection and pachytene occur.

Direct effects on CO rates are another possibility. However, predicting how CO rates respond to both stress and changing evolutionary forces (e.g. drift, selection) can be difficult [48,103]. In general, CO rates increase due to stress [1,18], though there are many possible mechanisms whereby stress may impact realized recombination rates. For example, recent work has shown that transposon activity also increases under stress [104,105]. Because this activity targets silencing machinery [106], a decrease in the accessibility to recombination proteins could explain an indirect change to recombination rates [107], which has been documented for other types of gene regulation [108]. Supporting the idea that genome accessibility may drive the connection between stress and recombination rate differences, early *Drosophila* studies showed broadly (greater than 5 Mb resolution) that centromeric and telomeric regions are more susceptible than the rest of the genome to change in recombination rate due to heat stress [24,109]. For example, Grell [109] observed a 36-fold increase due to heat in the centromeric region of chromosome 2. In many ways, a molecular understanding of how stress impacts meiotic recombination may distinguish between possible mechanisms indicated by regional and temporal studies.

In summary, there are many possible mechanisms and work in multiple systems that supports both direct and indirect processes as possible causes of recombination rate plasticity. However, the majority of studies we review here point to early meiotic events being the most susceptible to perturbation by stressful environments. This finding from *Drosophila* extends to studies in other systems, including *Sordaria*, liver wort, grasshopper and green algae, which also suggest early meiotic causes for plasticity in recombination, concurrent with the time that recombination occurs (discussed in [85,86]). Nonetheless, extending recombination plasticity work into other systems is likely to yield additional insights. For example, studies using organisms with holocentric chromosomes and inverted meiosis [79] could clarify if the MI and MII divisions have specific relationships to changes in the realized recombination rate. These and other species with unique meiotic features hold much promise for this area of research.

While the majority of work discussed here has found an increase in recombination rate in response to stress, work in both *Drosophila* [26] and tomatoes [18] found that age has the opposite effect, suggesting perhaps that age-related plasticity is due to other unknown processes. While the role of different environmental stressors, differences among organisms, and evolution of recombination rate plasticity have been reviewed extensively elsewhere [1,18,101,103,110], some groups have studied unique features of plasticity not explored in *Drosophila* that are worth highlighting here. For example, in *C. elegans*, sex-specific responses to stress exposure have been observed [30]. Studies have further shown that social stress in mice influences recombination rates [36]. While the fold-changes from these studies largely match those in *Drosophila* (up to threefold), some plant studies have observed up to a sevenfold-change in recombination in response to pathogen exposure [27].

## 4. Guidelines for studying recombination rate plasticity to uncover mechanisms

As outlined above, there have been a variety of experimental approaches for studying variation in recombination rate plasticity. This collective work in *D. melanogaster* and other taxa has pointed to a variety of possible early meiotic mechanisms for how experimental treatments could induce a change in recombination rate. Here, we outline four major guidelines for future work in this area aimed at distinguishing between mechanisms. First, many studies use mutant markers that are physically distant on the chromosome, only allowing broad-scale inferences of recombination rate differences. Here, we recommend using closer-spaced markers to uncover fine-scale differences in recombination rate. Second, many previous studies collect time-course recombination rates, which can be useful for pinpointing when during meiosis these changes occur. We recommend this study design as well, but note that it requires an understanding of the timing of events in gametogenesis, meiosis and recombination for the selected system. Third, the majority of studies simply compare differences in recombination rate across a specific region or sets of regions, but we encourage the use of other measures of recombination rate differences that can more precisely determine how the distribution of crossovers along the genome is changing. Finally, very few studies have accurately modelled variation using a robust statistical framework, such as a mixed-model approach, which we recommend here.

For each of the guidelines outlined below, we present results from an empirical experiment we conducted in *Drosophila pseudoobscura*. Recombination rate plasticity has not been studied in this species; therefore, it is a representative test of our experimental guidelines for non-model taxa. For our experiment, we chose the crossing scheme to match a previously published fine-scale genome-wide recombination study in *D. pseudoobscura* [58]. In our genetic cross we used two sequenced strains [111] in this species, Flagstaff 14 and Flagstaff 16, where F_1_ females were backcrossed to males from Flagstaff 16 to assay progeny CO rates. This control cross was conducted at 18°C. We repeated this cross in flies reared at a higher temperature (23°C) to assay the impact of heat stress, which matches experiments where the treatment is applied continuously during development as discussed above. To ensure the heat stress treatment resulted in stress on the organism, we measured differences in fecundity between the control temperature of 18°C and the stress temperature of 23°C and found a significant difference (*p* = 0.003, see electronic supplementary material). Detailed methods can be found in the electronic supplementary material. Below, we refer to the results of this experiment in the context of our recommended guidelines. Building on the work initiated 100 years ago demonstrating recombination rate plasticity [22], we present our work and these guidelines to direct the next century of work in this area specifically focused on uncovering mechanisms in a variety of taxa.

### (a) Guideline 1. Measure fine-scale genome-wide recombination rate to distinguish between global versus local effects

Previous work conducted at broad scales using visible markers confirms significant genome-wide variation in plasticity [24,109], rather than a global increase in recombination rate [37]. While there is renewed interest in this topic [78,84], these studies have focused on small regions of the genome where visible genetic markers allow rapid screens of recombinant progeny (but see [37]). To further refine our understanding of the factors contributing to variation in the genome-wide distribution of plasticity, we recommend studies use more fine-scale markers and markers spaced evenly across the genome. While we recognize that this guideline may lead to specific challenges in terms of cost, it also leads to several benefits including allowing non-model organisms to be used and avoiding fitness consequences of mutant markers [112]. Most studies reviewed here and elsewhere have been conducted through the utility of mutant visible markers that are broadly located throughout the genome. To assay recombination rate at a fine-scale, genotyping using molecular markers becomes necessary, which, while previously applied to estimating recombination rates in *Drosophila* [14,49,50,58], is more expensive than mutant screens. Because the term ‘fine-scale’ has various meanings, it is important to consider the relevance to the experimental goal. For example, to distinguish between global versus local chromosomal effects, markers approximately 1–2 Mb would be necessary, given they are also evenly spaced along the chromosome. However, to examine associations with particular genes to get at specific mechanisms, a scale of less than 100 kb would be required. Another interesting approach would be to introduce mutant markers via CRISPR in non-model taxa [45,46] to collect preliminary data about plasticity in a new system. Further, combination approaches using visible or genetically engineered markers for pre-screening and then continuing with fine-scale work [113] also hold much promise for non-model taxa.

Here, we have designed single nucleotide polymorphism (SNP) markers for the Sequenom platform [114], which allows multiplexing of 30–40 markers [43]. This allowed us to space markers evenly along a single chromosome, providing much more fine-scale plasticity results. Our mean marker distance here was 1.23 Mb. This approach and other recently described genotype-by-sequencing approaches [42,44] lend themselves well to non-model systems and the use of detecting crossovers in admixed populations using ancestry informative markers [81]. These latter approaches would be ideal for collecting the requisite fine-scale data that would be necessary for uncovering specific genes related to mechanism.

**Figure 3.**
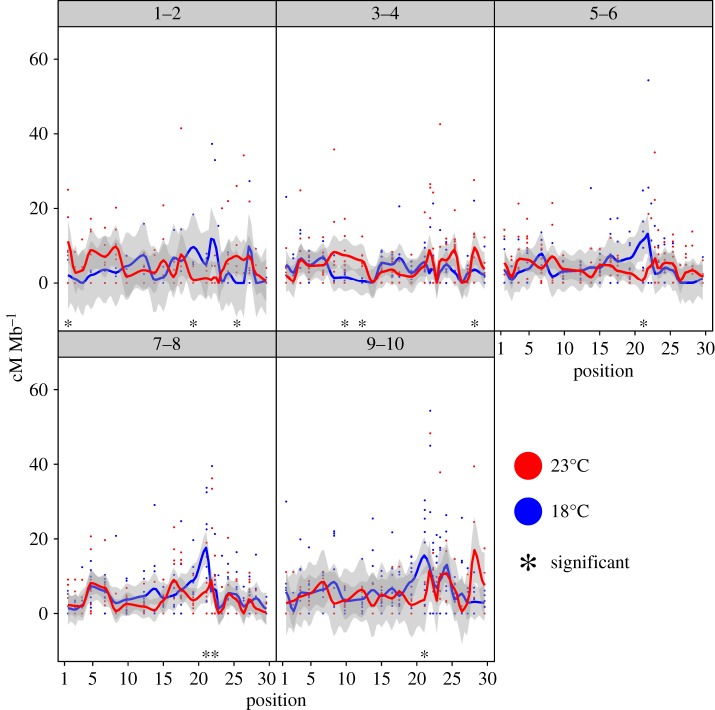
Here, we present our empirical results of recombination plasticity due to temperature in *D. pseudoobscura*. In all panels, blue indicates the 18°C results and red represents the 23°C results. Asterisks indicate significant least-squares means contrasts (see electronic supplementary material, figure S1). These graphs show raw point measurement data for recombination rate calculated from individual F_1_ parents, were LOESS smoothed using the two surrounding positions, and are presented with standard error of the smoother in grey, representing the variability across replicates. Each panel in the figure represents the 48 h time period during which each recombination rate measurement was estimated.

In figure 3, we show the results of our fine-scale study along the second chromosome of *D. pseudoobscura*. The fine-scale approach yields insights into regions that have higher recombination rates at multiple temperatures. Not only does this match previous work showing that some regions are more sensitive to recombination rate plasticity, but it also shows that the high-temperature treatment is not always higher than the control temperature. In fact, contrary to some studies, ours indicates that the converse also occurs in later time periods.

### (b) Guideline 2. To help pinpoint meiotic mechanisms, measure changes over time using time-course data

As highlighted in the selected *D. melanogaster* studies, measuring the change in recombination rate due to various treatments in a temporal context can elucidate various mechanisms, especially in the case of continuous treatments (figure 2). There are several challenges with regards to this aspect of experimental design. One is that splitting the total sample size into days dramatically reduces the overall power to distinguish between control and treatment (although see Guideline 4 (section 4d) for incorporating treatment and day together into a better modelling framework). Another caveat is that partitioning the offspring into vials based on maternal age is easy in a system such as flies, but in a non-model system this may be more difficult. Finally, time may not be a perfect indicator of meiotic timing. Specifically, there may be individual variance for meiotic progression that leads to variation in a ‘day’ effect. As mentioned above, in *D. melanogaster* experiments, such as done by Grell, a major advancement was to synchronize female oogenesis for temporal analysis. Here, we have chosen a different model specifically for the purpose of synchronized oogenesis. *D. pseudoobscura* females have synchronization of oogenesis across ovarioles, which allows time to be a better indicator of progression through oocyte development [115]. This synchronicity, either imposed within the experimental design or inherent in the study organism, is key to matching the time measurements with mechanisms based on meiotic timing.

In figure 3, we show the results of our study, specifically highlighting the differences between treatments by day. We collected progeny in 48 h increments. To avoid sample size issues and experimental complexity, one could design an experiment in wider intervals to first narrow down a specific time point of ‘sensitivity’ in recombination rate plasticity. Then, a more targeted collection in that period at more frequent intervals could be conducted. Here, we initially set up one large experiment where we collected greater than 12 000 progeny (see electronic supplementary material). We then down-sampled individuals for the first round of genotyping. Thus, to select more samples in the targeted ‘sensitive’ period, we could use samples from the original experiment, rather than conducting another experiment. This control led for variance due to block effects. Alternatively, one could collect very precise day information and bin multiple time points together initially to narrow the sensitive period, which would allow for flexibility with regard to sample size and statistical power. Here, we did not collect samples more frequently than at 48 h intervals, and so to further refine this time period, we would need to conduct at least one more targeted experiment.

### (c) Guideline 3. Incorporate a variety of measures of recombination rate differences

Owing to variation in when and how recombination is altered due to stress, it is important that studies consider not only measurements of absolute changes in recombination, but also changes in the overall distribution of COs. Rather than a random distribution along the genome, placement of COs along chromosomes has been shown to be controlled at three levels: (i) CO assurance ensures at least one CO per chromosome arm, (ii) CO homeostasis ensures the proportion of COs to non-COs resulting from double-strand breaks is maintained, and (iii) CO interference leads to reduced CO occurrence near existing COs [116,117]. CO assurance or the ‘obligatory crossover rule’ is simple to measure and has been observed in many species from yeast to mammals [116–118]. CO homeostasis has been measured by looking at rates of gene conversions associated with non-crossovers (NCOs) when compared with COs. This analysis requires precise NCO measurements, which is also experimentally challenging [50]. These measures range from 4 : 1 to 15 : 1 with conversion events much more likely than COs [119,120]. CO interference has been demonstrated to vary across different organisms [39,121–125]. Because of multiple levels of control, predicting responses of CO rates to both environmental and evolutionary forces (e.g. drift, selection) is difficult [48,103]. While many studies have focused on alterations in CO rates, several have also found changes in the distribution of CO events to be important. First, a recent human study found age-related plasticity is explained by a breakdown in CO interference [39]. Second, recombination rate is less concentrated into recombination hotspots (therefore more evenly distributed spatially along the genome) in dogs, yeast and birds, all of which are missing the key CO control protein, PRDM9 [126–128]. Third, *Drosophila* mutants of key meiotic genes have shown changes in the spatial distribution of COs [74]. Interestingly, several of these latter proteins are also involved in the process of interchromosomal effect [73,74]. In the Grell work discussed earlier, CO interference was measured and the plasticity results due to heat stress were compared with results from heterokaryotype experiments [109]. This showed that although changes in estimated crossover rates across the major chromosomes were fairly similar, CO interference was different in the heat treated versus heterokaryotype, except on the X chromosome. Therefore, measuring CO distribution is key to distinguishing between possible mechanisms of these two very different processes.

In our study, we measured CO control in two ways—CO interference and CO assurance. We did not measure CO homeostasis because NCOs have a narrow size distribution (approx. 300 bp) and require large sample sizes of approximately 1 million progeny [129]. Therefore, we did not have the statistical power to precisely localize NCO events or detect significant differences in the ratio of these events to COs in this experiment. For CO interference, we found that the 18°C treatment has a quick decline in interference as more distant markers are compared (xoi and qtl R packages; [130,131]). However, the 23°C treatment maintains a high level of CO interference to 25 cM (electronic supplementary material, figure S2). Our test for differences in CO assurance was not statistically different (*p* = 0.94). It is worth noting here that the adherence of different taxa, including *Drosophila*, to the obligatory crossover rule is not well studied, but seems to be quite variable within and between species [132]. We found that only approximately 75% of individuals in our study adhered to this rule.

### (d) Guideline 4. Adhere to a robust statistical model framework

As has been done previously in studies comparing recombination rates across experimental treatments [133,134], we recommend a more robust and comprehensive statistical framework be employed when analysing results on recombination rate plasticity. In the guidelines we discuss above, the experimental designs become quite complex, and independent comparisons of day and region do not properly account for multiple testing. Additionally, these studies typically include many crosses to get large sample sizes, which use multiple replicate females. However, most studies do not account for this source of variation. In fact, most studies report sample size in reference to the number of progeny rather than the number of F_1_ parents. Based on the guidelines of experimental design we suggest above, the experimental analysis needs to be equally adjusted to account for the sources of variation appropriately. We do not recommend using simple summary statistics, or independent tests (e.g. *χ*^2^). To appropriately account for non-independence of day and region, we suggest the use of mixed models. With regards to experimental design, we suggest being mindful that increased experimental complexity necessitates increased sample size for power to detect treatment differences (as mentioned in Guideline 2 (section 4b) above). While we suggest using mixed models to correctly account for dependence due to experimental design, it is important to recognize that model significance does not always implicate plasticity as the source of that variation. For example, in Priest *et al.* [133], it was noted that significance due to region had more to do with underlying heterogeneity in recombination rate and not variance due to their treatment (see below). Finally, mixed effects models are broadly applicable [135], and R implementation makes them easily accessible to researchers.

In our study, we estimated recombination in pseudo-replicate F_1_ females, and accounted for parental dependence with our suggested model framework. This represents an important improvement over previous studies because it accounts for variability due to experimental design, and strengthens inference about mechanisms controlling recombination rate plasticity. Generally, we cannot assume independence of a specific female F_1_ in a backcross experimental design. Each source vial for F_1_ female parents is considered a true replicate, while each female within a replicate is considered a pseudo-replicate. This non-independence presents a problem when constructing models of recombination rate because model assumptions (i.e. error independence) are not satisfied as a result of recombination rate estimates being ‘nested’ within parents, which affects model inferences. In order to ensure robust inferences regarding the relationship of treatment and recombination rate, a modelling strategy that appropriately accounts for non-independence among pseudo-replicate F_1_ females within a replicate is required. Specifically, we investigated the interaction of time (day), temperature and genomic position (fixed effects) on recombination rate (cM Mb^−1^), accounting for replicate (random effect) using a mixed effects model (lme4 R package; [136]). We compared the model with an intercept and random effect null model with a likelihood ratio test (table 1). Finally, following a significant likelihood ratio test, we tested least-squares means contrasts between 18 and 23 degrees at marker positions along the genome within day (lsmeans R package; [137], see [138] for a similar use). A subset of regions and days were significant in our study (see next section and electronic supplementary material).

**Table 1.** ANOVA table of mixed model of chromosome 2 showing significance of fixed effects. These results were produced using LmerTest with Saitherwaites approximation for degrees of freedom. *Italic values represent significance at 0.05.

	sum of squares	mean square	numerator d.f.	denominator d.f.	*F*	P*
position	215.99	9.39	23	1459.89	8.9	*<0*.*001*
temperature	0.04	0.04	1	4.27	0.04	0.85
day	24.97	6.24	4	1448.68	5.92	*<0*.*001*
position × temperature	61.32	2.67	23	1459.89	2.53	*<0*.*001*
position × day	103.23	1.12	92	1459.89	1.06	0.32
temperature × day	9.56	2.39	4	1448.68	2.27	0.06
position × temperature × day	95.51	1.04	92	1459.89	0.98	0.52

## 5. Preliminary findings of *D. pseudoobscura* plasticity experiment

Although our empirical results are provided here as an example of how to implement the proposed guidelines for continued studies in recombination rate plasticity, they also provide novel insights into plasticity in this species. First, these results represent the first evidence of recombination rate plasticity in *D. pseudoobscura*, despite a century of evidence in *D. melanogaster* and other species. Second, our model shows a significant effect of chromosomal position on recombination rate plasticity. This is due to documented heterogeneity in recombination broadly across the genome, which has been extensively reported elsewhere in this organism [14,49,58], close relatives [50], and other organisms [40]. Although this variation is not pertinent to our experiment, accounting for this known variation allows for better interpretation of the role that our treatment played in contributing to changes in recombination rate. More pertinent perhaps is that we observe a significant interaction term between temperature and position (table 1). This noted position significance is similar to other studies that have used a model-based statistical analysis of these types of results [133]. Third, we note a significant day component of variation in our experiment, which is consistent with early studies [139]. Further, as with position, we note non-significance for the interaction term between temperature and day (*p* = 0.06), which indicates that our treatment may not impact the proportion of recombinant progeny deposited per day.

Our study also presented several challenges worth noting here. First, the significance of interaction terms in a statistical model can be difficult to interpret, but lsmeans contrasts provide the ability to investigate interactions at combinations of experimental factors of interest more precisely (see starred regions in figure 3). This post hoc test is similar to Tukey's honestly significant difference (HSD) test after a significant ANOVA (see electronic supplementary material). Specifically, we see more significant regions in early (days 1–4) versus late (days 5–10) time points (figure 3). Further, five of six of these regions have higher recombination rate in the high-temperature treatment (electronic supplementary material, table S1), consistent with other studies of recombination rate plasticity due to temperature [99,140]. For the late time period, we see a consistent increase in recombination in the control temperature treatment at position 20.5 Mb (electronic supplementary material, table S1). This reduced recombination peak also corresponds to a region with twofold lower recombination rate when compared with the outgroup *Drosophila miranda* [58]. Interestingly, some of these regions overlap with regions of known higher recombination rate in this same cross [58]. This could point to either increased power in regions of higher recombination rate or an association between recombination rate and likelihood of plasticity. Further work with much larger sample sizes would be needed to distinguish between these scenarios.

Another challenge in our study is the overall low sample size used. Because we split our data further into multiple time points, each time point had less power to detect statistical differences in recombination rate. Specifically, each time point had between 140 and 321 individuals. Therefore, with a background recombination rate of 3.8 cM Mb^−1^, we only had power to detect large changes in recombination (approx. two- to threefold) in each interval. To increase power, we aggregated the early (1–4 days, *N* = 396) and late (5–10 days, *N* = 841) time points to increase overall sample size in each time interval. These results were largely similar to figure 3, suggesting that these findings are not artefacts of low sample size. The overall lack of power is further supported by electronic supplementary material, figure S1, which illustrates that we had several non-significant lsmeans estimates. Of course the biological relevance of these results requires additional samples for verification. We therefore consider this a preliminary analysis of plasticity in this species and plan to increase our sample size and further refine regions of increased plasticity in future studies.

## 6. Concluding remarks

All organisms experience heterogeneity in their environment, which impacts their evolution. Because recombination produces novel haplotypes, the influence of the environment on the evolution of species should account for the direct relationship between the environment and recombination rate. There are still many open questions in the study of recombination rate plasticity. Beyond variation in experimental approaches reviewed here, there are also considerable differences among study designs in the type and severity of the stress applied. Further, there is known variation in how an individual responds to various types of stress, which has not been thoroughly examined. Many recent studies target evolution of stress response networks in a variety of organisms, meaning this too is a new avenue to incorporate into studies on recombination rate plasticity. Individual stress response has been documented to vary considerably and could be associated with variation in recombination rate plasticity across studies. Many studies ignore the underlying genetic variation within a population for response to stress.

Interestingly, conflicting results have been reported for some sources of stress across organisms [1,18,37]. This suggests that there are as yet unknown sources of variation in plasticity and possible variation in mechanism depending on the organism, the treatment, or other sources. Further, different regions of the genome may be impacted differently owing to a variety of epigenetic and other genetic differences. Therefore, it is important to more thoroughly explore these sources of variation to interpret both positive and negative findings on recombination rate plasticity. Our experimental guidelines, together with recent advances in genotyping, suggest that this is a ripe area of research in the current age of genomics.

## Supplementary Material

Supplementary Methods

## Data accessibility

This article has no additional data.
