# Supplementary material for: Recombination rate plasticity: revealing mechanisms by design
Source: Philos Trans R Soc Lond B Biol Sci. 2017 Nov 6;372(1736):20160459. doi: 10.1098/rstb.2016.0459 (PMC5698621; doi:10.1098/rstb.2016.0459)
Supplement: Supplementary Methods [file rstb20160459supp1.docx]

Laurie Stevison, Stephen Sefick, Chase Rushton, and Rita Graze. Recombination rate plasticity: revealing mechanisms by design.

**Supplementary Methods**

Here we present the detailed cross design and statistical analysis of our empirical work in *Drosophila pseudoobscura*. As outlined in the main paper, we chose the crossing scheme to match a previously published fine-scale genome-wide recombination study in *D. pseudoobscura* (1). The genetic cross used two sequenced strains (2) in this species, Flagstaff 14 and Flagstaff 16, where F_1_ females were backcrossed to males from Flagstaff 16 to assay progeny CO rates. We repeated this cross in flies reared at a higher temperature to assay the impact of heat stress.

Because *D. pseudoobscura* has a longer egg-to-adult time period than *D. melanogaster*, we aged females 5 days prior to mating for them to reach full sexual maturity. Based on evidence of the known temperature ranges of this species, we reared flies at 23^o^C to maintain levels of fecundity needed for the experiment. To maximize differences in developmental times (3), we selected a control temperature of 18^o^C. We controlled for added stress of multiple mating by conducting single pair matings of F_1_ females because of the known impact on female recombination rates (4-6). We subsequently discarded males after 24 hours to prevent remating and any additional harassment. Further, to partition eggs based on female age, we transferred mated females to fresh food every 48 hours for a total of 10 days post-mating. We collected >12,000 progeny, but selected a subset of only 700 from each treatment genome-wide which correspond to three replicate families each for further genotyping. Among all progeny, we tested for a difference in F_1_ female fecundity based on treatment and found that the 18 degree treatment had a significantly higher fecundity (p=0.0003), suggesting that the higher temperature females had a lower fitness due to the extreme temperature.

To assay recombination rate in the progeny, we designed 35 multiplexed Sequenom (7) genotype markers. Based on the genomes discussed above, we aligned raw reads to the UCSC dp4 version of the genome, and called variants through the GATK best practices workflow (8). We hard-filtered variants following GATK recommendations. Briefly, we inspected annotation plots to arrive at hard filtering thresholds modifying thresholds based on GATK recommendations. We used Fisher strand bias <2, mapping quality <40, a test for mapping quality position (MQRankSum) <-12.5, and a test for read position (ReadPosRankSum) <-8. As a result of this analysis, we developed an R package “genotypeR” to facilitate marker design, quality control/quality assurance, recombination rate, and other analyses related to genotyping data (9). This software will be publically available on github upon publication of the associated manuscript (<https://github.com/StevisonLab/genotypeR>; doi: 10.5281/zenodo.801482).

Our designed markers cover roughly 20% of the *D. pseudoobscura* genome. This includes the whole 2nd chromosome (30 Mb), and a 6 Mb region on the XL, which was included to determine the future utility of a mutant strain with visible markers spanning this region. Only results from chromosome 2 markers are discussed in the accompanying manuscript. Following marker design we conducted genotyping on the Sequenom Platform (7),

For all analyses described unless otherwise stated, we used the R language for statistical computing (10). COs were counted between markers from raw Sequenom genotype data (7). Missing data points from instrument ‘No Calls’ required distributing COs among intervals proportional to physical distance. For example, assume we have three markers where a CO occurred between marker 1 and 3, with no genotype information for marker 2. In order to assign the CO to a region, we distributed the CO proportional to interval distance among the M1-M2 and M2-M3 intervals. Finally, the number of COs per individual, proportion of COs in an interval, and standardized measures of recombination rate (centimorgans per megabase (cM/Mb)) were calculated. In order to summarize data to investigate crossover assurance, we used a python script to count crossovers.

Following this, we investigated the interaction of time, temperature, and genomic position (fixed effects) on recombination rate (cM/Mb) accounting for the family random effect using a mixed effects model (lme4 R package; 11). Residuals were examined for deviations from linear assumptions, and we ln(cM/Mb+1) transformed the data to satisfy linear model assumptions. Following this, we compared the model with an intercept and random effect NULL model with a likelihood ratio test. Following a significant finding, we tested least squares means contrasts between 18 and 23 degrees at marker positions along the genome within day (Table S1) (lsmeans R package; 12). In this way, we were able to make inferences about recombination rate at differences in day, position, and temperature (fixed effects) for all "possible" families (random effect) (Figure S1).

## Supplementary References

1. McGaugh SE, Heil CS, Manzano-Winkler B, Loewe L, Goldstein S, Himmel TL, et al. Recombination modulates how selection affects linked sites in Drosophila. PLoS biology. 2012;10(11):e1001422. PubMed PMID: 23152720. Pubmed Central PMCID: 3496668.

2. McGaugh SE, Noor MA. Genomic impacts of chromosomal inversions in parapatric Drosophila species. Philosophical transactions of the Royal Society of London Series B, Biological sciences. 2012 Feb 5;367(1587):422-9. PubMed PMID: 22201171. Pubmed Central PMCID: 3233717.

3. Kuntz SG, Eisen MB. Drosophila embryogenesis scales uniformly across temperature in developmentally diverse species. PLoS genetics. 2014 Apr;10(4):e1004293. PubMed PMID: 24762628. Pubmed Central PMCID: 3998915.

4. Redfield H. Delayed mating and relationship of recombination to maternal age in *Drosophila melanogaster*. Genetics. 1966;53(3):593-607. PubMed PMID: ISI:A19667391400018. English.

5. Stevison LS. Male-mediated effects on female meiotic recombination. Evolution; international journal of organic evolution. 2012 Mar;66(3):905-11. PubMed PMID: 22380449.

6. Priest NK, Roach DA, Galloway LF. Mating-induced recombination in fruit flies. Evolution; international journal of organic evolution. 2007 Jan;61(1):160-7. PubMed PMID: ISI:000244285000014. English.

7. Gabriel S, Ziaugra L, Tabbaa D. SNP genotyping using the Sequenom MassARRAY iPLEX platform. Current protocols in human genetics / editorial board, Jonathan L Haines [et al]. 2009 Jan;Chapter 2:Unit 2 12. PubMed PMID: 19170031.

8. DePristo MA, Banks E, Poplin R, Garimella KV, Maguire JR, Hartl C, et al. A framework for variation discovery and genotyping using next-generation DNA sequencing data. Nature genetics. 2011 May;43(5):491-8. PubMed PMID: 21478889. Pubmed Central PMCID: 3083463.

9. Sefick S, Castronova, M, Stevison, L. genotypeR: an integrated R package for SNP genotype marker design and data analysis. In revision.

10. Kong A, Barnard J, Gudbjartsson DF, Thorleifsson G, Jonsdottir G, Sigurdardottir S, et al. Recombination rate and reproductive success in humans. Nature genetics. 2004 Nov;36(11):1203-6. PubMed PMID: WOS:000224832800024. English.

11. Bates D, Machler M, Bolker BM, Walker SC. Fitting Linear Mixed-Effects Models Using lme4. J Stat Softw. 2015 Oct;67(1):1-48. PubMed PMID: WOS:000365981400001. English.

12. Lenth RV. Least-Squares Means: The R Package lsmeans. J Stat Softw. 2016 Jan;69(1):1-33. PubMed PMID: WOS:000373914900001. English.

**Table S1.** Mixed model results of lsmean contrasts from our study of recombination rate plasticity in *D. pseudoobscura*. Only significant contrasts are shown, as well as overall model significance.

| **Likelihood Ratio*P*** | **start (Mb)** | **Day** | **estimate** | **SE** | **df** | ***T*** | ***P*** |
| --- | --- | --- | --- | --- | --- | --- | --- |
| < 2.2e-16 | 1.00 | a | 1.45 | 0.73 | 1406.14 | 1.98 | 0.048 |
|  | 18.00 | a | -1.96 | 0.73 | 1406.14 | -2.69 | 0.007 |
|  | 24.73 | a | 1.43 | 0.73 | 1406.14 | 1.97 | 0.049 |
|  | 8.58 | b | 1.12 | 0.56 | 1295.02 | 2.00 | 0.046 |
|  | 11.02 | b | 1.48 | 0.56 | 1295.02 | 2.64 | 0.008 |
|  | 27.66 | b | 1.33 | 0.56 | 1295.02 | 2.38 | 0.017 |
|  | 20.46 | c | -1.86 | 0.53 | 1261.14 | -3.47 | 0.001 |
|  | 20.46 | d | -1.22 | 0.49 | 1187.53 | -2.48 | 0.013 |
|  | 22.01 | d | -1.08 | 0.49 | 1187.53 | -2.20 | 0.028 |
|  | 20.46 | e | -1.21 | 0.61 | 1346.43 | -1.98 | 0.048 |

**Figure S1.** Least squares means contrast results at unique combinations of Position, Temperature, and Day. The point represents the lsmean and is bracketed by the standard error. Red represents significant differences in 23˚C minus 18˚C where the contrast is in the 23˚C direction. Blue represents significant differences in 23˚C minus 18˚C where the contrast is in the 18˚C direction. Grey represents no significant difference.

**Figure S2.** Crossover interference along chromosome 2 comparing the effect of temperature on coefficient of interference (COI). Blue indicates the 18°C results and red represents the 23°C results.
